# Geographic variation in *Dendrocalamus latiflorus* culm physical and mechanical traits is associated with source-climate gradients

**DOI:** 10.3389/fpls.2026.1946733

**Published:** 2026-10-14

**Authors:** Ying Fang, Hang Tao, Jiannan Lin, Jiaying Liu, Yawen Yang, Yushan Zheng, Liguang Chen, Jundong Rong, Tianyou He

**Affiliations:** 1 College of Forestry, Fujian Agriculture and Forestry University, Fuzhou, China; 2 College of Landscape Architecture and Art, Fujian Agriculture and Forestry University, Fuzhou, China

**Keywords:** bamboo biomechanics, bioclimatic variables, culm functional traits, Dendrocalamus latiflorus, geographic source, germplasm evaluation, partial least squares regression

## Abstract

**Introduction:**

Geographic variation in bamboo culm traits can reveal associations between source environments and biomechanical performance and can help prioritize germplasm for further testing.

**Methods:**

We quantified 11 physical and mechanical traits in 31 geographic sources of *Dendrocalamus latiflorus* from nine provincial-level regions of China and related source-level means to 25 geographic and climatic variables. Analyses combined coefficients of variation, Pearson correlations with Benjamini–Hochberg false-discovery-rate correction, partial least squares regression (PLSR) with leave-one-out cross-validation and 1,000 bootstrap resamples, Moran’s I tests of residual spatial autocorrelation, principal component analysis, objectively validated Ward clustering, and a seven-trait entropy-weighted TOPSIS evaluation with equal-weight sensitivity analysis.

**Results:**

Trait coefficients of variation ranged from 7.1% to 24.4%. After correction of 100 Pearson tests, 32 associations remained significant at *q* < 0.05; the strongest was between precipitation of the warmest quarter (BIO18) and compressive strength parallel to grain (CSPG; *r* = 0.658, *q* = 0.0058). PLSR showed the highest cross-validated performance for CSPG (Q^2^ = 0.599), moderate performance for air-dry and oven-dry density, and weak performance for modulus of rupture (Q^2^ = 0.076). Raw density and CSPG values were spatially autocorrelated, but PLSR residuals were not. Two validated trait classes showed no significant differences in six key source-climate variables. Entropy-weighted TOPSIS ranked P6, P4, and P19 highest; equal weighting produced strongly concordant ranks (Spearman rho = 0.911).

**Discussion:**

These results identify robust climate-associated trait patterns and candidate sources for further testing while avoiding causal inference from source-site sampling.

## 1 Introduction

Bamboo culms combine low mass with high axial strength and stiffness, so density, dimensional stability, and mechanical performance are important both as plant structural traits and as determinants of raw-material quality (Chave et al., 2009; Pérez-Harguindeguy et al., 2013). These properties arise from the hierarchical arrangement of fibers, vascular bundles, and parenchyma across the culm wall (Liese, 1987; Gibson, 2012; Dixon and Gibson, 2014). Functionally graded architectures allow bamboo to combine stiffness, strength, and damage tolerance at relatively low density (Amada et al., 1997; Wegst et al., 2015; Gao et al., 2022). Consequently, geographic variation in culm properties is relevant to functional plant ecology, germplasm evaluation, and the development of bamboo resources for engineered products (Sun et al., 2020; Chaowana et al., 2021; Xu et al., 2025).

*Dendrocalamus latiflorus* is a large sympodial bamboo widely cultivated in subtropical southern China for both edible shoots and culm utilization. Domestic provenance research first demonstrated practical gains from multi-trait selection in two Fujian provenance trials (Zheng, 2005). More recently, a 20-provenance survey across nine provincial-level regions documented substantial variation in growth, crown, and leaf phenotypes and reported correlations with geographic and climatic factors (Li et al., 2023). These studies established a clear provenance-evaluation foundation in China, but they did not quantify the integrated physical-mechanical performance of mature culms across the same broad environmental space. Studies of *D. latiflorus* further show that culm age and position influence density, strength, and single-fiber mechanics (Wang et al., 2014; You et al., 2017), while recent work demonstrates radial variation in vascular-bundle architecture within Dendrocalamus, including *D. latiflorus* (Y. Chen et al., 2024). Across other bamboo species, geographic variation in anatomy and mechanical performance can be substantial (Rini et al., 2023), and detailed structural studies continue to link vascular-bundle distribution and fiber organization to strength and stiffness (Zhang et al., 2024). Together with evidence that management can modify culm structure and performance in large sympodial bamboos (Yan et al., 2024), this literature underscores the need to separate structural, developmental, management, and source-environment contributions to culm material traits.

Climate may be associated with culm properties through effects on carbon balance, water relations, tissue maturation, and cell-wall formation. Temperature regulates plant metabolic rates and the timing of vascular-tissue development, whereas water availability influences cell expansion, assimilate transport, and hydraulic function (Fonti et al., 2010; Rossi et al., 2007; Poorter et al., 2010). At broad spatial scales, stem density covaries with anatomical, hydraulic, and mechanical functions (Hacke et al., 2001; Zanne et al., 2010; Lachenbruch and McCulloh, 2014), and multiple thermal and hydrological dimensions are associated with wood density (Mo et al., 2024). For *D. latiflorus* itself, recent habitat-suitability modeling shows that climate, soil, vegetation, and elevation jointly structure its geographic distribution in China (L.-J. Chen et al., 2024), reinforcing the need to avoid treating macroclimate as an isolated source-site driver. Provenance trials in trees similarly demonstrate that climate-density relationships are species dependent and require common-environment testing to separate inherited differentiation from plasticity (Nabais et al., 2018; You et al., 2021). For bamboo, the absence of secondary vascular growth does not eliminate post-elongation maturation, but the specific physiological pathways linking source climate to mature culm properties remain insufficiently measured.

The present study extends earlier work in four ways. First, it evaluates 31 geographic sources spanning nine provincial-level regions, providing broad cross-province coverage. Second, it jointly analyzes all 11 measured culm physical and mechanical traits rather than focusing on a single property. Third, it strengthens inference by combining Benjamini-Hochberg false-discovery-rate correction, cross-validated and bootstrap-stabilized PLSR, an explicit residual spatial-autocorrelation test, and objective cluster validation. Fourth, it broadens germplasm prioritization from four equally weighted performance variables to seven measured criteria that include dimensional stability, applies entropy-based objective weighting, and evaluates ranking sensitivity under equal weights.

Here, provenance denotes a geographic source population. Because culms were sampled at their source locations rather than in a common garden, the observed differences may reflect inherited differentiation, phenotypic plasticity, soils, stand history, management, and other spatially structured factors. We therefore interpret all climate results as source-environment associations rather than causal climatic effects or evidence of genetic adaptation (Savolainen et al., 2007; Alberto et al., 2013). We asked: (1) how strongly do 11 culm physical and mechanical traits vary among the 31 sources; (2) which source-climate variables remain associated with density and mechanical performance after multiplicity control and multivariate stability assessment; (3) whether residual spatial dependence remains after PLSR; (4) whether validated multivariate trait classes correspond to discrete climatic differences; and (5) which sources show high composite material performance under a seven-criterion TOPSIS framework and whether those rankings are sensitive to weighting. The study is intended to generate testable ecological hypotheses and prioritize material for subsequent common-garden and multi-environment validation.

## 2 Materials and methods

### 2.1 Provenance locations and climatic data

Culm material was collected in 2023 from 31 geographic source locations distributed across nine major *D. latiflorus*-producing provincial-level regions of China. Longitude, latitude, and elevation were recorded in the field using a handheld GPS receiver. Long-term bioclimatic variables BIO1-BIO19 were obtained from WorldClim version 2.1 at 2.5 arc-min resolution (approximately 4.5 km at the equator) and represent the 1970–2000 climatic normal (Fick and Hijmans, 2017). Extreme minimum temperature, extreme maximum temperature, and mean annual sunshine duration were obtained from regional meteorological data sources for a comparable climatological reference period.

These climatic variables were used as long-term descriptors of source-site environmental regimes, not as reconstructions of weather in the 2023 sampling year or the approximately three-year developmental history of each culm. **Supplementary Table S1** lists provenance coordinates, elevation, and the principal site-climate descriptors used for interpretation; **Supplementary Table S2** contains the complete 25-variable geographic and climatic predictor matrix. This distinction between climatic normal and developmental-period weather was maintained throughout the revised interpretation.

### 2.2 Plant material and sampling

Sampling was conducted from May to September 2023 in Fujian, Guangxi, Guangdong, Sichuan, Guizhou, Yunnan, Zhejiang, Jiangxi, and Chongqing. Source locations followed a grid-based design and were separated by more than 50 km. Within plantations or naturally occurring pure stands with broadly comparable stand structure and management history, three spatially separated bamboo clumps were selected per provenance, and one dominant, straight, disease-free, 3-year-old culm was sampled from each clump. One intact internode segment was collected at breast height (1.3 m) from each culm, retaining the complete outer-to-inner culm-wall structure.

The selected clumps were physically distinct and showed no visible rhizome connections in the field. On this basis, the three sampled culms were operationally treated as representing three independent genets and were used as three biological replicates for trait measurement. Genet identity was not confirmed using molecular markers; therefore, independence should be understood as field-based rather than genetically verified. D. latiflorus is widely cultivated and is not listed as a nationally protected plant in China. Access was coordinated with local forest or plantation managers, and species identity was confirmed in the field by researchers from the College of Forestry, Fujian Agriculture and Forestry University using diagnostic morphological characters.

### 2.3 Measurement of physical and mechanical properties

Air-dry density, oven-dry density, radial, tangential, and volumetric shrinkage, moisture content, compressive strength parallel to grain, modulus of rupture (MOR), and modulus of elasticity (MOE) were determined according to GB/T 15780–1995 and ISO 22157:2019 (GB/T, 1995; ISO, 2019). Mechanical tests were conducted using a WDW electronic universal testing machine (DANS, Shenzhen New Sansi, Shenzhen, China). Bending specimens measured 160 mm × 10 mm × t mm and compression specimens measured 20 mm × 20 mm × t mm, where t was culm-wall thickness. The loading rate was 30 mm min−1. Specimens were conditioned for 3 months at 21 °C and 70% relative humidity; test moisture content was approximately 12%, and mechanical properties are reported at approximately 12% moisture content. The calculation equations are shown in Equations 1–9:

(1) $$\text{ADD}=\frac{m_{w}}{V_{w}}$$

(2) $$\text{ODD}=\frac{m_{0}}{V_{0}}$$

(3) $$W=\frac{\left(m_{w}-m_{0}\right)}{m_{0}}\times 100\%$$

(4) $$\text{ADRS}/\text{ODRS}=\frac{\left(R_{s}-R_{d}\right)}{R_{s}}\times 100\%$$

(5) $$\text{ADTS}/\text{ODTS}=\frac{\left(T_{s}-T_{d}\right)}{T_{s}}\times 100\%$$

(6) $$\text{ADVS}/\text{ODVS}=\frac{\left(V_{s}-V_{d}\right)}{V_{s}}\times 100\%$$

(7) $$\text{CSPG}=\frac{P_{max}}{A}$$

(8) $$MOR\;=\frac{3P_{max}L}{2b{\text{h}}^{2}}$$

(9) $$MOE\;=\frac{\Delta PL^{3}}{4b{\text{h}}^{3}f}$$

where ADD and ODD are air-dry and oven-dry density, respectively; mw and m0 are air-dry and oven-dry mass; Vw and V0 are the corresponding specimen volumes; W is moisture content; ADRS/ODRS, ADTS/ODTS, and ADVS/ODVS are radial, tangential, and volumetric shrinkage; Rs, Ts, and Vs are the radial dimension, tangential dimension, and volume in the water-saturated state; Rd, Td, and Vd are the corresponding values in the air-dry or oven-dry state; CSPG is compressive strength parallel to grain; Pmax is the maximum failure load; A is the loaded area; L is the support span; b and h are specimen width and height; ΔP is the load increment within the elastic range; and f is the corresponding deflection.

### 2.4 Statistical analysis

Data were analyzed in R version 4.4.2 and Python. Provenance means, standard deviations, and coefficients of variation (CVs) were calculated for each trait. Normality and homogeneity of variance were assessed using Shapiro–Wilk and Levene tests, respectively, and provenance differences were evaluated by one-way ANOVA followed by Duncan’s multiple-range test at P < 0.05. Pearson correlations were calculated between 25 geographic/climatic variables and four focal traits: air-dry density (ADD), oven-dry density (ODD), compressive strength parallel to grain (CSPG), and modulus of rupture (MOR). Benjamini–Hochberg false-discovery-rate (BH-FDR) correction was applied across all 100 tests, with q < 0.05 considered significant. Predictors were Z-score standardized before multivariate analyses. Separate partial least squares regression (PLSR) models were fitted for ADD, ODD, CSPG, and MOR. Model complexity and performance were evaluated by leave-one-out cross-validation (LOOCV) using Q² and RMSECV, and stability was further assessed by 1,000 bootstrap resamples. Predictor importance was summarized by the median variable importance in projection (VIP), its 95% bootstrap interval, and the frequency of VIP > 1 (Wold et al., 2001). Spatial dependence was evaluated using Moran’s I for both raw traits and PLSR residuals based on a four-nearest-neighbor spatial weights matrix, with significance assessed by 9,999 permutations. Principal component analysis (PCA; Jolliffe and Cadima, 2016) and Ward hierarchical clustering were performed using the 11 standardized culm traits. Candidate cluster solutions (k = 2–6) were evaluated using silhouette, Calinski–Harabasz, and Davies–Bouldin indices, and climatic differences between the retained classes were tested using Wilcoxon rank-sum tests. Composite material performance was evaluated using TOPSIS based on seven traits, with density and mechanical properties treated as benefit criteria and volumetric shrinkage as cost criteria. Entropy-derived weights were used for the primary analysis, and an equal-weight TOPSIS analysis was conducted as a sensitivity test; ranking agreement was assessed using Spearman correlation (Hwang and Yoon, 1981).

## 3 Results

### 3.1 Variation in culm traits among geographic sources

Across the 31 sources, CVs of the 11 physical and mechanical traits ranged from 7.1% to 24.4% (**Supplementary Table S3**). Air-dry tangential shrinkage showed the highest CV (24.4%), followed by MOE (21.7%), air-dry radial shrinkage (17.1%), air-dry volumetric shrinkage (16.6%), oven-dry tangential shrinkage (14.4%), and oven-dry volumetric shrinkage (12.6%). MOR and CS had CVs of 11.2% and 9.8%, respectively, whereas air-dry and oven-dry density had the lowest CVs, 7.1% and 8.3%. Thus, density varied less among source populations than shrinkage and stiffness.

P27 had the highest air-dry density (0.97 g cm−3), oven-dry density (0.92 g cm−3), and CS (64.95 MPa). P24 had the highest MOR (167.26 MPa), whereas P4 had the highest MOE (19,327.93 MPa). P12 combined high shrinkage with the lowest MOE. These contrasts show that density, strength, stiffness, and dimensional stability were not synchronized across geographic sources. A unified comparison of selected high-performing sources is provided in **Supplementary Table S4**.

### 3.2 FDR-corrected associations between source climate and culm traits

Among the 100 Pearson tests, 50 had nominal P < 0.05 before multiplicity correction. After BH-FDR correction, 32 associations remained significant at q < 0.05 and six remained at q < 0.01. **Figure 1** shows 11 representative geographic and climatic variables used to summarize the principal thermal, topographic, and precipitation axes.

**Figure 1:**
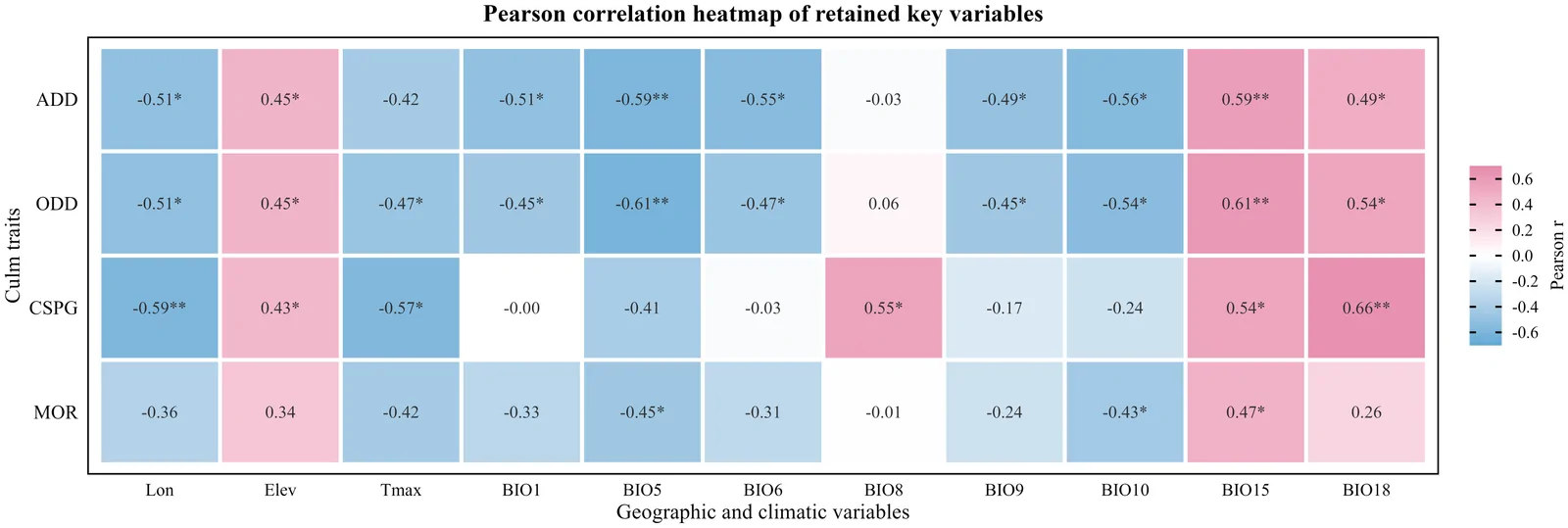
Pearson correlation heatmap between selected geographic and climatic variables and four culm traits of *Dendrocalamus latiflorus*. ADD, air-dry density; ODD, oven-dry density; CSPG, compressive strength parallel to grain; MOR, modulus of rupture. Cell values indicate Pearson correlation coefficients (*r*), with blue and pink representing negative and positive correlations, respectively. Asterisks indicate significance after Benjamini–Hochberg false-discovery-rate correction across all 100 tests: *q < 0.05 and **q < 0.01. Only the 11 retained keyvariables are shown.

BIO5 was negatively associated with ADD and ODD (r = −0.593 and −0.605, respectively; both q < 0.01) and with MOR (r = −0.454, q < 0.05). BIO15 was positively associated with ADD, ODD, CSPG, and MOR (r = 0.469-0.613), including q < 0.01 associations with both density traits. BIO18 was positively associated with ADD, ODD, and CSPG; the BIO18-CS relationship was the strongest correlation in the complete analysis (r = 0.658, q = 0.0058). Longitude was negatively associated with ADD, ODD, and CSPG, whereas elevation was positively associated with these traits. Extreme maximum temperature was negatively associated with ODD and CSPG, and BIO10 was negatively associated with ADD, ODD, and MOR. The pattern therefore reflected trait-specific associations with multiple thermal and hydrological dimensions rather than a single climatic axis.

### 3.3 PLSR performance and stability of important predictors

Cross-validated performance differed markedly among the four PLSR models (**Table 1**). The CS model had the highest Q² (0.599) with cumulative R²Y of 81.2%. The ODD and ADD models had Q² values of 0.374 and 0.333, respectively. In contrast, MOR had Q² = 0.076, and its bootstrap Q² median was −0.071, indicating that the calibration structure did not generalize reliably to omitted observations. Across 1,000 bootstrap resamples, the CSPG model retained a Q² median of 0.523 (0.322-0.644), whereas the uncertainty interval for MOR extended well below zero.

**Table 1:** Cross-validated performance and bootstrap stability of PLSR models for four culm traits.

Culm trait	Cumulative R²Y (%)	Q² (LOOCV)	RMSECV (LOOCV)	Bootstrap Q² median (P2.5–P97.5)	Bootstrap RMSECV median (P2.5–P97.5)	RMSECV/mean (%)
Air-dry density (ADD; g cm⁻³)	70.5	0.333	0.085	0.256 (0.001–0.437)	0.090 (0.078–0.104)	12.1
Oven-dry density (ODD; g cm⁻³)	70.7	0.374	0.076	0.305 (0.128–0.471)	0.080 (0.070–0.090)	11.3
Compressive strength parallel to grain (CSPG; MPa)	81.2	0.599	6.657	0.523 (0.322–0.644)	7.256 (6.272–8.655)	14.4
Modulus of rupture (MOR; MPa)	64.6	0.076	23.045	−0.071 (−0.609–0.189)	24.815 (21.600–30.412)	22.5

PLSR, partial least squares regression; LOOCV, leave-one-out cross-validation; RMSECV, root mean square error of cross-validation. Bootstrap intervals are the 2.5th and 97.5th percentiles from 1,000 resamples; n = 31 geographic sources.

Extreme maximum temperature was the most consistently high-importance variable across models (**Table 2**), with median VIP values of 1.32, 1.38, 1.44, and 1.54 for ADD, ODD, CSPG, and MOR and VIP > 1 frequencies of 90.9%, 94.9%, 97.6%, and 95.3%, respectively. BIO5, BIO15, and elevation also had relatively high and stable VIP values in the density models. In the CSPG model, BIO8 and BIO18 both had median VIP = 1.36; BIO18 exceeded VIP = 1 in 95.7% of bootstrap samples and was also the strongest FDR-corrected Pearson association. Although the MOR model identified several high-VIP variables, its low Q² means these variables should be treated as candidate association signals rather than stable predictive factors.

**Table 2:** Bootstrap-stabilized variable importance in projection (VIP) statistics for key climatic predictors of the four focal culm traits.

Culm trait	Key climatic predictor	Median VIP	95% CI	VIP > 1 frequency (%)	Pearson r
Air-dry density (ADD)	Extreme maximum temperature (°C)	1.32	0.82–1.76	90.9	−0.419
	BIO5: maximum temperature of warmest month (°C)	1.21	0.86–1.47	88.6	−0.593
	BIO15: precipitation seasonality	1.16	0.81–1.45	81.4	0.590
	Elevation (m)	1.15	0.85–1.42	83.5	0.453
	BIO10: mean temperature of warmest quarter (°C)	1.10	0.80–1.38	74.0	−0.561
Oven-dry density (ODD)	Extreme maximum temperature (°C)	1.38	0.91–1.81	94.9	−0.467
	BIO5: maximum temperature of warmest month (°C)	1.23	0.90–1.48	90.8	−0.605
	BIO15: precipitation seasonality	1.20	0.83–1.48	85.2	0.613
	Elevation (m)	1.17	0.88–1.43	86.1	0.445
	BIO18: precipitation of warmest quarter (mm)	1.15	0.87–1.44	83.4	0.539
Compressive strength parallel to grain (CSPG)	Extreme maximum temperature (°C)	1.44	1.00–1.80	97.6	−0.568
	BIO8: mean temperature of wettest quarter (°C)	1.36	0.74–2.04	86.3	0.550
	BIO18: precipitation of warmest quarter (mm)	1.36	0.96–1.78	95.7	0.658
	Extreme minimum temperature (°C)	1.23	0.62–1.91	75.1	0.406
	Longitude (°E)	1.20	0.92–1.47	92.0	−0.593
Modulus of rupture (MOR)	Extreme maximum temperature (°C)	1.54	0.89–2.02	95.3	−0.415
	Elevation (m)	1.21	0.82–1.54	85.4	0.339
	Extreme minimum temperature (°C)	1.14	0.78–1.55	76.9	0.052
	BIO8: mean temperature of wettest quarter (°C)	1.08	0.69–1.51	65.6	−0.007
	BIO5: maximum temperature of warmest month (°C)	1.05	0.76–1.37	63.0	−0.454

n = 31 geographic sources. Median VIP and 95% CI are based on 1,000 bootstrap resamples. VIP > 1 frequency is the percentage of bootstrap samples in which VIP exceeded 1. Pearson r is the unadjusted correlation coefficient; negative values indicate negative associations. CI, confidence interval.

### 3.4 Spatial autocorrelation of raw traits and PLSR residuals

Raw ADD, ODD, and CSPG showed significant positive spatial autocorrelation under the four-nearest-neighbor weights matrix, with Moran’s I values of 0.273 (P = 0.0068), 0.261 (P = 0.0085), and 0.336 (P = 0.0029), respectively; raw MOR was not significant (I = 0.125, P = 0.1143) (**Table 3**). In contrast, none of the PLSR residuals showed significant spatial autocorrelation: residual Moran’s I was −0.129 for AD (P = 0.3342), −0.127 for OD (P = 0.3293), −0.166 for CS (P = 0.1797), and −0.078 for MOR (P = 0.6686). The same qualitative result was obtained using three- through six-nearest-neighbor definitions. Thus, broad geographic structure was evident in several raw traits, but no residual spatial dependence was detected after the fitted PLSR structure. This does not establish climatic causation, but it reduces the likelihood that the reported PLSR associations are explained solely by unmodeled broad-scale spatial autocorrelation.

**Table 3:** Spatial autocorrelation of raw culm traits and PLSR residuals using a four-nearest-neighbor spatial weights matrix.

Culm trait	Raw-trait Moran’s I	P value	PLSR-residual Moran’s I	P value
Air-dry density (ADD)	0.273	0.0068	−0.129	0.3342
Oven-dry density (ODD)	0.261	0.0085	−0.127	0.3293
Compressive strength parallel to grain (CSPG)	0.336	0.0029	−0.166	0.1797
Modulus of rupture (MOR)	0.125	0.1143	−0.078	0.6686

Significance was assessed with 9,999 permutations. Raw ADD, ODD, and CSPG showed significant positive spatial autocorrelation, whereas none of the PLSR residuals were significant. Sensitivity checks using three- through six-nearest-neighbor definitions yielded the same qualitative conclusion. PLSR, partial least squares regression.

### 3.5 Principal component analysis and validated trait classes

PC1 and PC2 explained 47.2% and 31.1% of total variation, respectively, for a cumulative 78.3% (**Figure 2B**). MOE and MOR loaded mainly toward the negative side of PC1, whereas air-dry and oven-dry radial, tangential, and volumetric shrinkage loaded toward the positive side. Air-dry density, oven-dry density, and CSPG were oriented more strongly along PC2. The ordination therefore separated bending performance, dimensional change, and density/compression dimensions.

**Figure 2:**
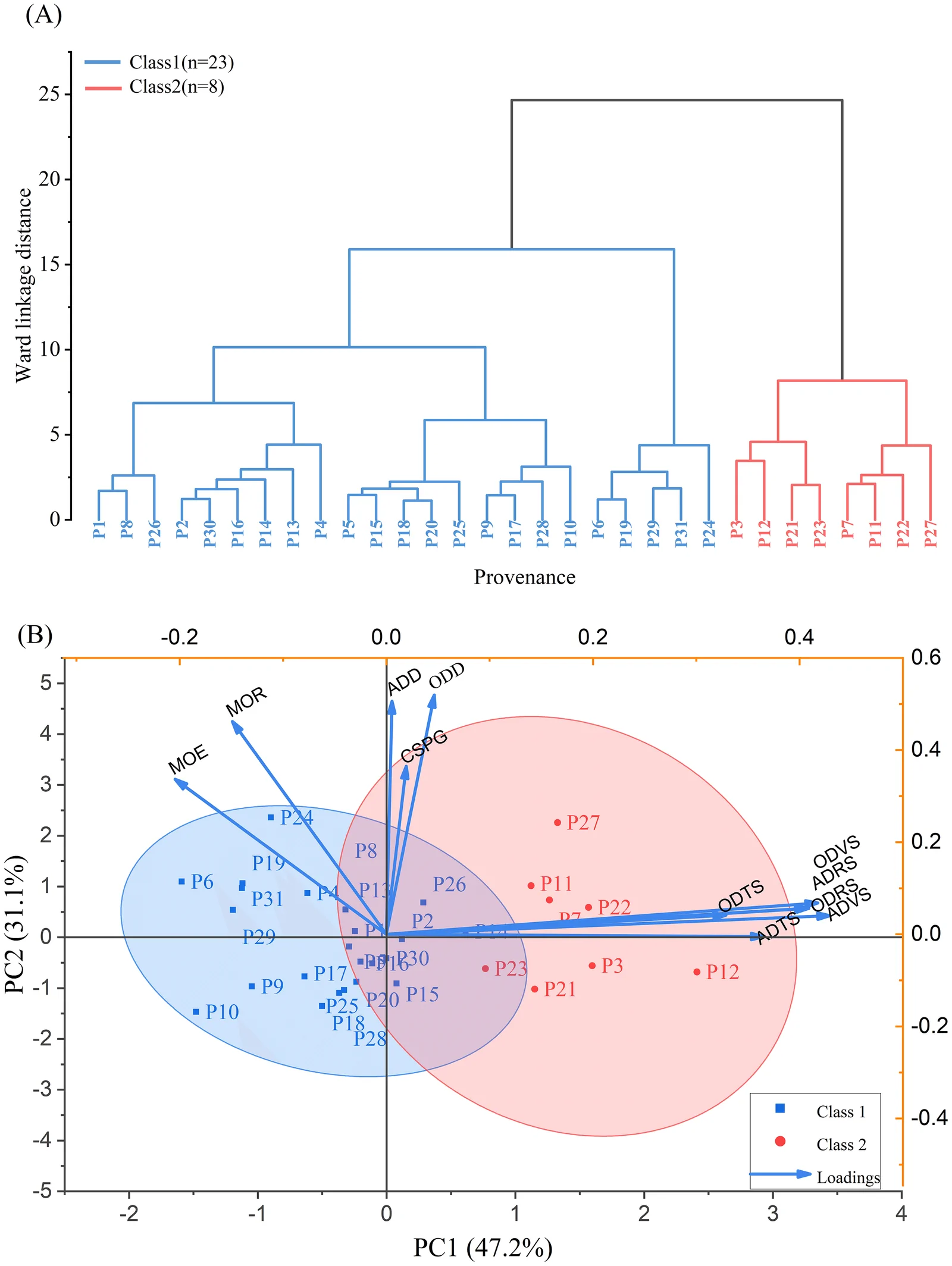
Ward hierarchical clustering and principal component analysis of 31 *Dendrocalamus latiflorus* provenances based on 11 standardized culm physical and mechanical traits. **(A)** Ward dendrogram showing two validated classes, with Class 1 (n = 23) in blue and Class 2 (n = 8) in red. **(B)** PCA biplot showing provenance scores and trait loadings. PC1 and PC2 explained 47.2% and 31.1% of the total variation, respectively. Shaded ellipses indicate the distributions of the two classes.

Objective validation favored two Ward clusters over k = 3-6 (**Supplementary Table S5**). Class 1 contained P1, P2, P4, P5, P6, P8, P9, P10, P13, P14, P15, P16, P17, P18, P19, P20, P24, P25, P26, P28, P29, P30, and P31 (n = 23). Class 2 contained P3, P7, P11, P12, P21, P22, P23, and P27 (n = 8). PCA and clustering were broadly concordant: Class 2 occurred mainly on the PC1-positive side near several shrinkage loadings, whereas Class 1 extended more broadly toward the MOR/MOE directions and the ordination center.

### 3.6 Source-climate characteristics of the two trait classes

Six source-environment variables were compared between the two trait classes (**Figure 3**). Wilcoxon rank-sum tests were not significant for BIO18 (P = 0.074), BIO5 (P = 0.086), BIO15 (P = 0.255), elevation (P = 0.255), extreme maximum temperature (P = 0.542), or BIO8 (P = 0.821). Descriptively, Class 2 had higher median BIO18 (639.5 versus 504.0 mm) and elevation (489.2 versus 190.7 m) and lower median BIO5 (29.25 versus 31.50 °C) than Class 1, but these tendencies did not meet the P < 0.05 criterion. The trait classes therefore did not map onto discrete climate categories within this dataset.

**Figure 3:**
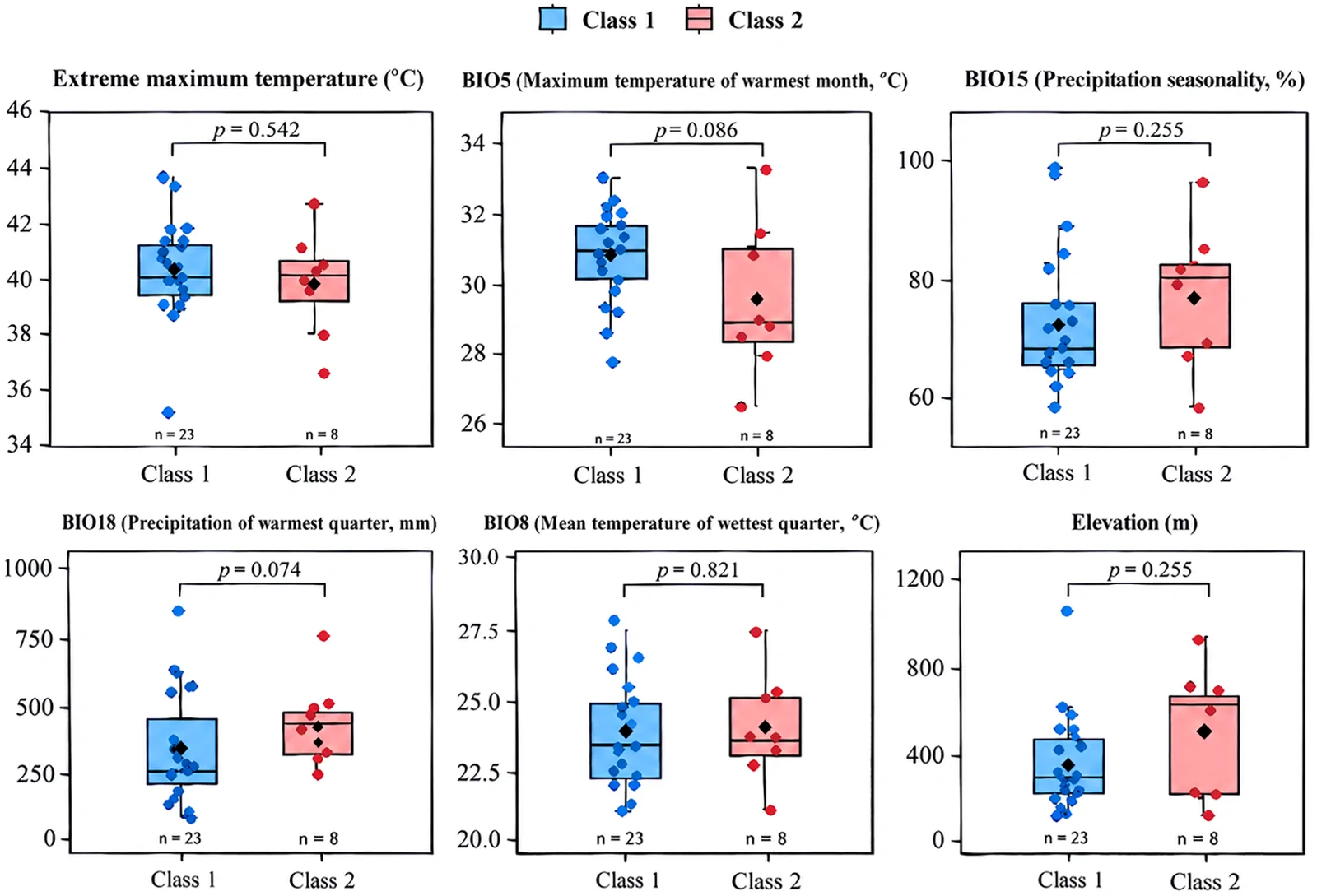
Comparison of six source-environment variables between Class 1 (n = 23) and Class 2 (n = 8), including extreme maximum temperature, BIO5 (maximum temperature of the warmest month), BIO15 (precipitation seasonality), BIO18 (precipitation of the warmest quarter), BIO8 (mean temperature of the wettest quarter), and elevation. P-values were obtained using Wilcoxon rank-sum tests. None of the between-class differences was statistically significant at P < 0.05.

### 3.7 Seven-criterion TOPSIS ranking and weight sensitivity

Entropy weighting assigned the largest weight to air-dry volumetric shrinkage (0.3384), followed by MOE (0.2191) and oven-dry volumetric shrinkage (0.1730); CSPG and MOR received weights of 0.0940 and 0.0986, and air-dry and oven-dry density received 0.0399 and 0.0370 (**Table 4**). These weights quantify information dispersion within the current dataset and should not be interpreted as universal biological or engineering importance.

**Table 4:** Entropy weights used in the seven-criterion TOPSIS evaluation of culm material performance.

Criterion	Entropy weight	Direction
Air-dry density (ADD)	0.0399	Benefit (higher is better)
Oven-dry density (ODD)	0.0370	Benefit (higher is better)
Compressive strength parallel to grain (CSPG)	0.0940	Benefit (higher is better)
Modulus of rupture (MOR)	0.0986	Benefit (higher is better)
Modulus of elasticity (MOE)	0.2191	Benefit (higher is better)
Air-dry volumetric shrinkage (ADVS)	0.3384	Cost (lower is better)
Oven-dry volumetric shrinkage (ODVS)	0.1730	Cost (lower is better)

Note: Benefit criteria were maximized and cost criteria were minimized. The entropy weights sum to 1.0000 and quantify information dispersion within the present dataset; they should not be interpreted as universal biological or engineering importance. TOPSIS, Technique for Order Preference by Similarity to Ideal Solution.

Entropy-weighted TOPSIS closeness coefficients ranged from 0.076 to 0.781 (**Figure 4**). P6 (Ci = 0.781), P4 (0.771), and P19 (0.739) ranked first to third, followed by P31, P29, P24, P10, and P9 within the upper quartile. P27 illustrates the tradeoff incorporated by the expanded framework: despite having the highest density and CSPG, its relatively high volumetric shrinkage reduced Ci to 0.351.

**Figure 4:**
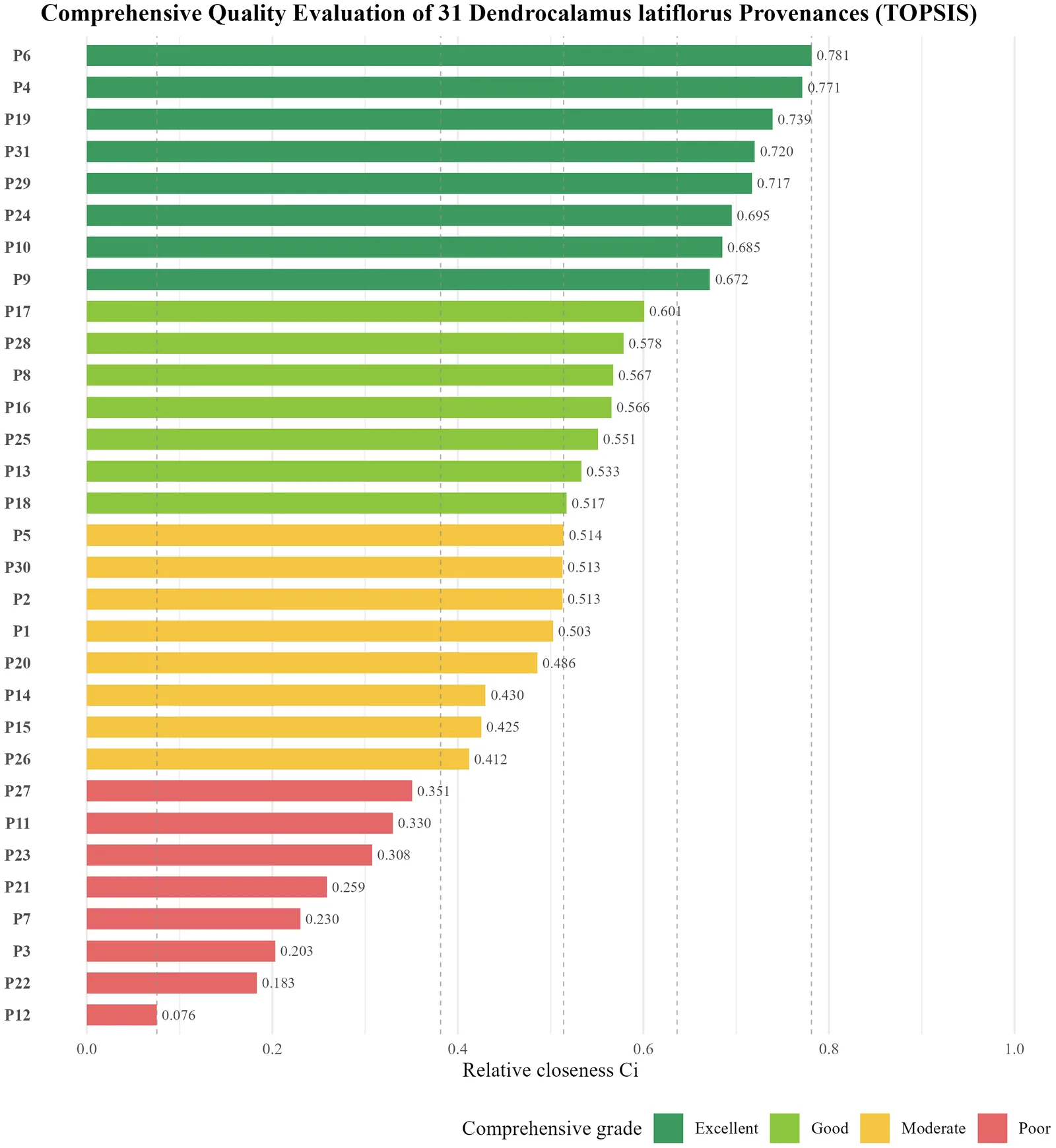
Entropy-weighted TOPSIS evaluation of comprehensive culm material performance across 31 *Dendrocalamus latiflorus* provenances. Horizontal bars show the relative closeness coefficient (*C*_i_) for each provenance and are colored according to comprehensive grade (excellent, good, moderate, or poor). Higher *C*_i_ values indicate higher composite performance under the seven-criterion evaluation framework.

Under the equal-weight sensitivity analysis, P24 ranked first, followed by P6, P4, P19, P31, P29, P8, and P10. The entropy-weight and equal-weight ranks were strongly concordant (Spearman rho = 0.911, P < 0.001), and seven of the eight entropy-weight upper-quartile sources remained in the equal-weight upper quartile (**Supplementary Table S6**). Thus, exact ordering depended on the weighting rule, but the high-priority candidate set was largely stable.

## 4 Discussion

### 4.1 Geographic-source differentiation and multidimensional culm performance

All 11 measured culm traits varied among the 31 geographic sources, with CVs of 7.1%-24.4%. The magnitude of variation is sufficient to identify contrasting source materials, but the source-site design does not allow the differences to be assigned to genetic provenance effects alone. Instead, the observed contrasts integrate inherited variation, phenotypic plasticity, soils, stand history, and management. Provenance-trial evidence in trees shows that the balance between genetic control and environmental plasticity in density-related traits is species dependent (Nabais et al., 2018), and geographic pattern by itself does not establish local adaptation (Savolainen et al., 2007; Alberto et al., 2013).

The lower CVs of density than of shrinkage and MOE also emphasize the multidimensional nature of bamboo material performance. Culm density depends on the amount and spatial distribution of vascular bundles, fibers, and parenchyma (Liese and Tang, 2015), whereas mechanical behavior additionally reflects fiber-bundle architecture, radial grading, and cell-wall hierarchy (Nogata and Takahashi, 1995; Dixon and Gibson, 2014; Habibi et al., 2015; Li and Shen, 2011). Crack propagation, fiber maturation, and fiber fraction further influence stiffness, strength, and toughness (Gibson, 2012; Habibi and Lu, 2014; Wang et al., 2012; Shao et al., 2010). The contrasting profiles of P27, P24, P4, and P12 therefore support multi-trait evaluation rather than assuming that a single source will maximize density, strength, stiffness, and dimensional stability simultaneously.

### 4.2 Climate-trait associations after multiplicity, stability, and spatial checks

The revised analyses provide three complementary safeguards against overinterpretation: BH-FDR correction of the complete Pearson test family, bootstrap-stabilized PLSR, and residual spatial-autocorrelation testing. After FDR correction, 32 of 100 associations remained significant, indicating that a substantial subset of the pairwise signals was not explained by multiplicity alone. However, the directions were trait specific. BIO5 and other thermal variables were mainly negatively associated with density and MOR, whereas BIO15 was positively associated with all four focal traits, and BIO18 showed the strongest positive association with CSPG. The data therefore do not support a simple statement that one climatic dimension ‘drives’ culm properties.

PLSR adds a multivariate perspective because climatic predictors are strongly collinear. The repeated importance of extreme maximum temperature across bootstrap samples suggests that warm-end thermal conditions participate consistently in the latent environmental structure, while BIO18 and BIO8 contribute strongly to the CSPG model. A Pearson coefficient describes a marginal pairwise association, whereas PLSR VIP describes contribution within a collinear latent-predictor system; disagreement between the two is therefore expected and should not be treated as mechanistic evidence. The weak MOR Q² is especially important: high-VIP variables in that model do not have reliable out-of-sample predictive support and are best regarded as hypotheses for future testing.

Temperature and water availability can plausibly influence carbon assimilation, respiration, developmental timing, and cell-wall processes (Fonti et al., 2010; Rossi et al., 2007; Poorter et al., 2010). Bamboo fibers also undergo wall thickening and lignification after elongation (Murphy and Alvin, 1997; Lybeer and Koch, 2005; Wang et al., 2012). The spatial test further qualifies inference. ADD, ODD, and CSPG were positively spatially structured in the raw data, as expected for source traits measured across a broad geographic range. No significant Moran’s I remained in PLSR residuals under the primary four-neighbor definition or the three- to six-neighbor sensitivity checks. This result indicates that the fitted multivariate structure captured the broad spatial pattern detectable by these weights. It does not remove confounding by unmeasured spatially structured variables such as soils or management, and it therefore strengthens statistical adequacy without converting association into causation.

### 4.3 Trait classes reflect continuous gradients rather than discrete climate zones

PCA and objectively validated Ward clustering summarized the 11-trait space as two major classes. PC1 primarily contrasted bending performance with dimensional-change traits, which explains why some dense or strong sources could still occur in the shrinkage-oriented Class 2: clustering was based on the complete multivariate phenotype rather than a single target trait.

None of the six selected source-climate variables differed significantly between classes. BIO18 and BIO5 showed suggestive but non-significant tendencies, and Class 2 had higher median elevation and BIO18 and lower BIO5, but the evidence was insufficient to define discrete ecotypes. This distinction is important. Continuous-variable analyses can detect trait-specific environmental gradients even when an unsupervised trait classification does not correspond to sharply separated climatic groups. Microtopography, soil properties, management, and the small and unequal class sizes may further weaken one-to-one correspondence between trait classes and macroclimate.

### 4.4 Germplasm prioritization, expanded criteria, and ranking sensitivity

The revised TOPSIS framework incorporates five benefit criteria and two dimensional-stability cost criteria. Under entropy weighting, P6, P4, and P19 were the highest-ranked sources, whereas P24 remained within the upper performance class because of its very high MOR. The low composite score of P27 despite exceptional density and CSPG shows why shrinkage cannot be ignored when the objective is balanced material performance.

Entropy weights reflect variation within this particular dataset rather than universal engineering value. Consequently, sensitivity to weighting is essential. Equal weighting changed the exact order, placing P24 first, but the overall ranks remained strongly correlated and seven of eight upper-quartile sources were retained. This agreement suggests that the candidate set is more stable than any single rank position. The analysis still excludes unmeasured traits highlighted by the reviewers, including culm biomass yield, natural durability, processability, and economic performance. The resulting ranking should therefore be used for candidate prioritization rather than immediate regional deployment. Common-garden and multi-environment trials are needed to test trait stability and genotype-by-environment interaction before breeding or cultivation recommendations are made (Aitken and Bemmels, 2016).

### 4.5 Limitations and future directions

The principal limitation is the *in-situ* source-site sampling design. Climate is confounded with geographic position, soil physicochemical properties, nutrient status, stand density, silvicultural history, microtopography, and other local factors. For each source, one culm was sampled from each of three spatially separated, physically distinct clumps with no visible rhizome connections, and the culms were therefore treated as three independent genets and biological replicates. However, genet identity was not verified using molecular markers, so a residual possibility of within-source clonal relatedness cannot be completely excluded. These constraints prevent separation of inherited differentiation from environmental plasticity and management effects.

A second limitation is temporal scale. WorldClim 1970–2000 normals and regional climatological summaries describe long-term source environments but do not reproduce meteorological conditions in 2023 or the sequence of extreme events experienced during shoot emergence, culm maturation, and hardening over the preceding approximately three years. Extreme heat, drought, storms, and seasonal anomalies during that developmental window could affect mature culm properties but were not reconstructed. Likewise, soil moisture and microclimate were not measured directly.

Third, the source-level sample size (n = 31) is modest relative to 25 highly correlated environmental predictors. PLSR, LOOCV, bootstrap stability assessment, FDR control, and residual Moran’s I testing improve statistical discipline but do not replace independent external validation. The MOR model illustrates this limitation clearly because its Q² was near zero and its bootstrap median was negative. Class 2 contained only eight sources, limiting power for between-class climate comparisons.

Future work should establish replicated common-garden and multi-site trials under standardized management, genotype source populations to quantify genetic structure and clonality, and evaluate genotype-by-environment interaction (Savolainen et al., 2007; Alberto et al., 2013; Aitken and Bemmels, 2016). Environmental models should incorporate soil physicochemical properties, nutrients, stand density, management history, microtopography, and directly measured microclimate. Anatomical, chemical, and physiological measurements—including vascular-bundle density, fiber proportion, fiber-wall thickness, cellulose organization, lignin content, photosynthesis, and water status—would then allow source-environment associations to be linked to explicitly tested mechanisms.

## 5 Conclusion

The 31 D. latiflorus geographic sources showed clear multidimensional variation in 11 culm physical and mechanical traits (CV = 7.1%-24.4%). After BH-FDR correction, 32 of 100 source-environment associations remained significant, with BIO18 and CS showing the strongest correlation. PLSR provided its best cross-validated performance for CSPG, moderate performance for density, and weak performance for MOR. Raw density and CSPG were spatially structured, whereas fitted PLSR residuals showed no significant residual Moran’s I. Objective cluster validation supported two trait classes, but the classes did not differ significantly in six key source-climate variables, consistent with continuous rather than discrete environmental gradients. A seven-criterion entropy-weighted TOPSIS framework ranked P6, P4, and P19 highest, and the candidate set was largely retained under equal weighting. These results identify climate-associated phenotypic patterns and candidate material for further testing, but they do not demonstrate climatic causation or inherited adaptation. The *in-situ* design, temporally mismatched climatic normals, unmeasured soil and stand covariates, field-based genet assignment without molecular confirmation, and limited sample size must be addressed through common-garden, multi-environment, genetic, anatomical, chemical, and physiological validation before regional deployment recommendations are made.

## Data Availability

The original contributions presented in the study are included in the article/**Supplementary Material**. Further inquiries can be directed to the corresponding author.
