# Supplementary material for: Geographic variation in Dendrocalamus latiflorus culm physical and mechanical traits is associated with source-climate gradients
Source: Front Plant Sci. 2026 Oct 14;17:1946733. doi: 10.3389/fpls.2026.1946733 (PMC13713850; doi:10.3389/fpls.2026.1946733)
Supplement: Supplementary file 1 [file SupplementaryFile1.docx]

**Supplementary Material**

This file contains Supplementary Tables S1-S3.

**Supplementary Table S1.** Geographic coordinates and selected climatic variables for 31 *Dendrocalamus latiflorus* Munro provenances sampled in China.

| **Provenance ID** | **Provenance location** | **Longitude** | **Latitude** | **Elevation (m)** | **Extreme Tmin (°C)** | **Extreme Tmax (°C)** | **BIO1 Annual mean temperature (°C)** | **BIO5 Maximum temperature of warmest month (°C)** | **BIO8 Mean temperature of wettest quarter (°C)** | **BIO10 Mean temperature of warmest quarter (°C)** | **BIO11 Mean temperature of coldest quarter (°C)** | **BIO15 Precipitation seasonality (CV, %)** | **BIO18 Precipitation of warmest quarter (mm)** | **BIO19 Precipitation of coldest quarter (mm)** |
| --- | --- | --- | --- | --- | --- | --- | --- | --- | --- | --- | --- | --- | --- | --- |
| P1 | Xixiangtang, Nanning, Guangxi | 107°59′26″E | 22°46′54″N | 126.8 | -1.9 | 38.9 | 22.3 | 32.7 | 28.6 | 28.6 | 14.5 | 71.8 | 770 | 126 |
| P2 | Xingning, Nanning, Guangxi | 108°21′01″E | 22°57′38″N | 209.2 | -3.5 | 39.3 | 20.9 | 31.3 | 27.5 | 27.5 | 13.0 | 67.2 | 727 | 149 |
| P3 | Jiedong, Jieyang, Guangdong | 116°20′16″E | 23°39′03″N | 102.5 | 0.2 | 39.7 | 21.2 | 31.3 | 27.3 | 27.3 | 13.9 | 61.6 | 660 | 144 |
| P4 | Longhai, Zhangzhou, Fujian | 117°40′08″E | 24°22′02″N | 93.7 | 0.4 | 40.9 | 20.3 | 31.4 | 22.5 | 26.9 | 13.1 | 60.4 | 419 | 125 |
| P5 | Nanjing, Zhangzhou, Fujian | 117°15′53″E | 24°22′52″N | 49.3 | -2.9 | 40.7 | 20.7 | 32.1 | 23.1 | 27.3 | 13.3 | 59.0 | 428 | 136 |
| P6 | Mangshi, Dehong, Yunnan | 98°32′34″E | 24°28′19″N | 982.4 | -2.1 | 34.9 | 20.1 | 29.0 | 24.2 | 24.2 | 14.0 | 97.9 | 991 | 50 |
| P7 | Xingyi, Qianxinan, Guizhou | 104°59′38″E | 24°51′38″N | 817.9 | -3.3 | 36.5 | 18.9 | 29.2 | 24.8 | 24.8 | 11.4 | 82.3 | 689 | 64 |
| P8 | Yongfu, Guilin, Guangxi | 109°58′48″E | 24°56′18″N | 136.6 | -2.9 | 39.4 | 19.3 | 32.2 | 23.2 | 27.6 | 10.0 | 71.4 | 736 | 213 |
| P9 | Hua'an, Zhangzhou, Fujian | 117°33′07″E | 25°00′54″N | 110.8 | -2.4 | 41.2 | 19.3 | 31.5 | 21.8 | 26.1 | 11.7 | 59.1 | 500 | 142 |
| P10 | Zhangping, Longyan, Fujian | 117°28′35″E | 25°02′16″N | 360.3 | -5.4 | 40.2 | 19.3 | 31.5 | 21.9 | 26.1 | 11.7 | 58.8 | 503 | 146 |
| P11 | Wangmo, Qianxinan, Guizhou | 106°09′22″E | 25°19′31″N | 579.9 | -2.5 | 40 | 15.8 | 26.7 | 22.5 | 22.5 | 7.7 | 78.2 | 665 | 63 |
| P12 | Luodian, Qiannan, Guizhou | 106°28′12″E | 25°20′24″N | 548.7 | -1.8 | 41.2 | 17.3 | 28.8 | 23.3 | 24.3 | 8.9 | 75.6 | 619 | 64 |
| P13 | Yongchun, Quanzhou, Fujian | 118°14′57″E | 25°21′08″N | 194.9 | -3.3 | 39.7 | 18.7 | 30.6 | 21.1 | 25.7 | 11.2 | 60.5 | 388 | 142 |
| P14 | Hanjiang, Putian, Fujian | 118°56′39″E | 25°39′06″N | 170 | -2.3 | 40 | 18.8 | 30.6 | 21.1 | 26.1 | 10.9 | 63.4 | 373 | 141 |
| P15 | Ganzhou, Jiangxi | 114°58′27″E | 25°47′21″N | 155.6 | -3.9 | 40 | 18.4 | 32.4 | 22.5 | 27.2 | 8.7 | 51.9 | 450 | 214 |
| P16 | Yongtai, Fuzhou, Fujian | 119°03′58″E | 25°52′43″N | 20 | -4.8 | 41.1 | 19.4 | 32.1 | 21.9 | 27.2 | 11.3 | 61.3 | 496 | 152 |
| P17 | Yong'an, Sanming, Fujian | 117°22′09″E | 25°55′36″N | 190.7 | -5.6 | 40.1 | 19.8 | 33.6 | 23.1 | 27.5 | 11.4 | 59.5 | 503 | 186 |
| P18 | Minhou, Fuzhou, Fujian | 119°10′19″E | 25°59′11″N | 120 | -2.2 | 41.5 | 19.7 | 32.5 | 22.1 | 27.6 | 11.5 | 60.7 | 371 | 156 |
| P19 | Sandu, Qiannan, Guizhou | 107°59′24″E | 25°59′17″N | 384.4 | -3.5 | 39.2 | 16.9 | 30.3 | 24.0 | 25.5 | 7.5 | 64.8 | 523 | 89 |
| P20 | Fu'an, Ningde, Fujian | 119°47′56″E | 26°51′23″N | 23 | -4.3 | 41.9 | 18.5 | 31.7 | 20.9 | 26.9 | 9.8 | 56.0 | 541 | 184 |
| P21 | Yanping, Nanping, Fujian | 118°10′53″E | 26°40′58″N | 89.9 | -9.8 | 40.5 | 19.7 | 33.9 | 22.9 | 27.9 | 10.8 | 64.0 | 556 | 204 |
| P22 | Cangnan, Zhejiang | 120°25′33″E | 27°31′06″N | 11.8 | -6.2 | 40.4 | 18.4 | 32.0 | 20.9 | 27.1 | 9.3 | 51.2 | 513 | 165 |
| P23 | Yanjin, Zhaotong, Yunnan | 104°11′24″E | 28°12′36″N | 452 | -1.3 | 42.8 | 16.4 | 29.3 | 24.6 | 24.6 | 7.2 | 78.5 | 550 | 52 |
| P24 | Suijiang, Zhaotong, Yunnan | 104°01′22″E | 28°35′32″N | 434.3 | -1.2 | 40.6 | 15.5 | 28.2 | 23.7 | 23.7 | 6.4 | 81.7 | 587 | 50 |
| P25 | Fushun, Zigong, Sichuan | 104°50′03″E | 29°05′57″N | 305.5 | -1.4 | 41.5 | 17.9 | 31.1 | 26.2 | 26.2 | 8.8 | 78.8 | 602 | 61 |
| P26 | Longchang, Neijiang, Sichuan | 105°17′39″E | 29°20′43″N | 364 | -2.2 | 40.1 | 16.9 | 29.7 | 24.7 | 25.0 | 7.8 | 86.8 | 736 | 61 |
| P27 | Hongya, Meishan, Sichuan | 103°15′13″E | 29°43′52″N | 526.3 | -4.2 | 37.9 | 15.9 | 28.3 | 23.3 | 23.9 | 6.9 | 94.8 | 870 | 61 |
| P28 | Fengdu, Chongqing | 107°42′22″E | 29°46′31″N | 400 | -0.6 | 43.9 | 16.3 | 30.9 | 23.4 | 25.3 | 6.6 | 62.3 | 501 | 64 |
| P29 | Beibei, Chongqing | 106°35′25″E | 29°51′32″N | 322.5 | -1.7 | 42 | 17.3 | 31.9 | 26.3 | 26.3 | 7.7 | 68.6 | 519 | 56 |
| P30 | Fuling, Chongqing | 107°30′32″E | 29°52′23″N | 85.9 | -1.3 | 43.5 | 18.1 | 32.9 | 25.2 | 27.1 | 8.5 | 63.2 | 504 | 64 |
| P31 | Dongpo, Meishan, Sichuan | 103°43′00″E | 30°12′21″N | 459.2 | -3.6 | 39.2 | 17.0 | 29.8 | 24.5 | 25.2 | 7.8 | 97.0 | 779 | 48 |

**Note.** Geographic coordinates are reported in degrees, minutes, and seconds. Tmin and Tmax denote extreme minimum and maximum temperatures, respectively. BIO variables follow the standard WorldClim bioclimatic-variable definitions; BIO15 is expressed as the coefficient of variation. Selected variables correspond to the climatic predictors evaluated in the main analyses.

**Supplementary Table S2.** Additional climatic variables for 31 *Dendrocalamus latiflorus* Munro provenances sampled in China.

| **Provenance ID** | **Provenance location** | **Mean annual sunshine duration (h)** | **BIO2 Mean diurnal range (°C)** | **BIO3 Isothermality (×100)** | **BIO4 Temperature seasonality (SD ×100)** | **BIO6 Minimum temperature of coldest month (°C)** | **BIO7 Temperature annual range (°C)** | **BIO9 Mean temperature of driest quarter (°C)** | **BIO12 Annual precipitation (mm)** | **BIO13 Precipitation of wettest month (mm)** | **BIO14 Precipitation of driest month (mm)** | **BIO16 Precipitation of wettest quarter (mm)** | **BIO17 Precipitation of driest quarter (mm)** |
| --- | --- | --- | --- | --- | --- | --- | --- | --- | --- | --- | --- | --- | --- |
| P1 | Xixiangtang, Nanning, Guangxi | 1554.6 | 7.7 | 33.2 | 584.5 | 9.5 | 23.2 | 16.0 | 1522 | 260 | 33 | 770 | 118 |
| P2 | Xingning, Nanning, Guangxi | 1980.7 | 7.1 | 30.8 | 598.2 | 8.4 | 22.9 | 14.7 | 1514 | 250 | 33 | 727 | 120 |
| P3 | Jiedong, Jieyang, Guangdong | 1899.3 | 7.3 | 32.7 | 558.1 | 9.0 | 22.3 | 15.7 | 1554 | 250 | 32 | 660 | 109 |
| P4 | Longhai, Zhangzhou, Fujian | 2069.4 | 7.7 | 33.3 | 574.0 | 8.2 | 23.2 | 15.1 | 1228 | 195 | 27 | 514 | 89 |
| P5 | Nanjing, Zhangzhou, Fujian | 1860.0 | 8.2 | 34.2 | 581.5 | 8.1 | 24.0 | 15.1 | 1319 | 211 | 29 | 561 | 100 |
| P6 | Mangshi, Dehong, Yunnan | 2330.5 | 11.5 | 48.8 | 427.5 | 5.4 | 23.7 | 14.0 | 1584 | 377 | 13 | 991 | 50 |
| P7 | Xingyi, Qianxinan, Guizhou | 1611.5 | 8.8 | 38.2 | 546.0 | 6.3 | 22.9 | 11.4 | 1257 | 241 | 16 | 689 | 64 |
| P8 | Yongfu, Guilin, Guangxi | 1417.0 | 7.4 | 27.6 | 726.0 | 5.3 | 26.9 | 11.9 | 1888 | 376 | 49 | 950 | 161 |
| P9 | Hua'an, Zhangzhou, Fujian | 1833.9 | 8.8 | 35.0 | 593.1 | 6.3 | 25.1 | 13.4 | 1337 | 217 | 27 | 590 | 105 |
| P10 | Zhangping, Longyan, Fujian | 1689.9 | 8.8 | 35.1 | 594.7 | 6.3 | 25.2 | 13.5 | 1354 | 220 | 28 | 598 | 109 |
| P11 | Wangmo, Qianxinan, Guizhou | 1480.8 | 7.6 | 32.3 | 598.6 | 3.0 | 23.6 | 7.7 | 1271 | 242 | 16 | 665 | 63 |
| P12 | Luodian, Qiannan, Guizhou | 1299.5 | 7.9 | 32.2 | 626.7 | 4.2 | 24.7 | 8.9 | 1235 | 233 | 17 | 622 | 64 |
| P13 | Yongchun, Quanzhou, Fujian | 1749.0 | 7.9 | 32.6 | 600.4 | 6.2 | 24.4 | 16.4 | 1331 | 229 | 27 | 595 | 105 |
| P14 | Hanjiang, Putian, Fujian | 1934.1 | 6.9 | 28.7 | 630.3 | 6.5 | 24.1 | 16.5 | 1312 | 250 | 27 | 597 | 98 |
| P15 | Ganzhou, Jiangxi | 1807.1 | 7.8 | 27.3 | 752.9 | 3.8 | 28.6 | 10.5 | 1507 | 240 | 41 | 640 | 158 |
| P16 | Yongtai, Fuzhou, Fujian | 1736.4 | 7.1 | 28.2 | 657.3 | 7.0 | 25.1 | 17.0 | 1320 | 250 | 32 | 596 | 110 |
| P17 | Yong'an, Sanming, Fujian | 1683.4 | 9.3 | 33.7 | 654.3 | 6.1 | 27.6 | 16.4 | 1501 | 255 | 40 | 700 | 145 |
| P18 | Minhou, Fuzhou, Fujian | 1727.2 | 6.9 | 27.5 | 665.7 | 7.3 | 25.3 | 17.3 | 1335 | 254 | 33 | 595 | 113 |
| P19 | Sandu, Qiannan, Guizhou | 1125.0 | 7.7 | 28.1 | 733.7 | 2.9 | 27.4 | 7.5 | 1214 | 214 | 26 | 572 | 89 |
| P20 | Fu'an, Ningde, Fujian | 1706.7 | 7.0 | 26.8 | 707.7 | 5.4 | 26.3 | 11.9 | 1624 | 273 | 44 | 639 | 139 |
| P21 | Yanping, Nanping, Fujian | 1592.6 | 8.6 | 30.7 | 699.8 | 5.8 | 28.1 | 12.4 | 1658 | 301 | 43 | 806 | 156 |
| P22 | Cangnan, Zhejiang | 1802.5 | 7.7 | 28.1 | 732.2 | 4.5 | 27.5 | 11.6 | 1497 | 215 | 39 | 550 | 154 |
| P23 | Yanjin, Zhaotong, Yunnan | 868.6 | 7.4 | 28.2 | 698.5 | 2.9 | 26.4 | 7.2 | 1041 | 193 | 16 | 550 | 52 |
| P24 | Suijiang, Zhaotong, Yunnan | 971.1 | 6.9 | 26.6 | 694.2 | 2.3 | 25.8 | 6.4 | 1085 | 218 | 15 | 587 | 50 |
| P25 | Fushun, Zigong, Sichuan | 1142.1 | 6.2 | 24.2 | 702.7 | 5.4 | 25.7 | 8.8 | 1146 | 221 | 19 | 602 | 61 |
| P26 | Longchang, Neijiang, Sichuan | 1339.7 | 6.7 | 25.8 | 693.0 | 3.9 | 25.7 | 7.8 | 1319.5 | 295.5 | 18.5 | 740.5 | 61 |
| P27 | Hongya, Meishan, Sichuan | 963.2 | 7.1 | 27.6 | 683.3 | 2.5 | 25.8 | 6.9 | 1493 | 370 | 18 | 879 | 61 |
| P28 | Fengdu, Chongqing | 1301.5 | 6.7 | 24.1 | 757.6 | 2.9 | 28.0 | 6.6 | 1238 | 183 | 18 | 532 | 64 |
| P29 | Beibei, Chongqing | 1036.8 | 6.5 | 23.4 | 747.3 | 4.3 | 27.6 | 7.7 | 1144 | 190 | 17 | 519 | 56 |
| P30 | Fuling, Chongqing | 1133.1 | 6.7 | 23.8 | 754.9 | 4.9 | 28.0 | 8.5 | 1214 | 182 | 19 | 526 | 64 |
| P31 | Dongpo, Meishan, Sichuan | 966.0 | 7.3 | 27.3 | 706.1 | 3.1 | 26.7 | 7.8 | 1328 | 322 | 13 | 800 | 48 |

**Note.** BIO variables follow the standard WorldClim definitions. BIO3 is calculated as BIO2/BIO7 × 100, and BIO4 is the standard deviation of monthly temperature multiplied by 100. Temperature and precipitation are expressed in °C and mm, respectively.

**Supplementary Table S3.** Culm physical and mechanical properties of 31 *Dendrocalamus latiflorus* Munro provenances.

| **Provenance ID** | **Modulus of elasticity MOE (MPa)** | **Modulus of rupture MOR (MPa)** | **Compressive strength parallel to grain CS (MPa)** | **Air-dry density** **ρ_w_ (g cm−3)** | **Oven-dry density** **ρ_0_ (g cm−3)** | **Air-dry tangential** **shrinkage** **β_1W_ (%)** | **Air-dry radial** **shrinkage** **β_2W_ (%)** | **Air-dry volumetric** **shrinkage** **β_W_ (%)** | **Oven-dry tangential** **shrinkage** **β_1_ (%)** | **Oven-dry radial** **shrinkage** **β_2_ (%)** | **Oven-dry volumetric** **shrinkage** **β_v_ (%)** |
| --- | --- | --- | --- | --- | --- | --- | --- | --- | --- | --- | --- |
| P1 | 8763.72 ± 1502.28cdef (17.1%) | 106.22 ± 6.67cdefghi (6.3%) | 64.41 ± 2.67ab (4.1%) | 0.67 ± 0.03defghijk (5.2%) | 0.63 ± 0.04defghijk (6.6%) | 3.48 ± 1.53bc (43.9%) | 12.03 ± 0.41bcdefg (3.4%) | 15.49 ± 1.61bcdefghi (10.4%) | 7.1 ± 1.75cd (24.7%) | 14.81 ± 0.57bcdefghij (3.8%) | 21.12 ± 2.21bcdef (10.4%) |
| P2 | 9673 ± 2298.66bcde (23.8%) | 101.07 ± 4.86defghij (4.8%) | 50.56 ± 9.09cde (18.0%) | 0.68 ± 0.03cdefghijk (5.1%) | 0.65 ± 0.03cdefghijk (5.0%) | 6.62 ± 0.29abc (4.4%) | 8.99 ± 0.87defghijk (9.6%) | 15.36 ± 0.76bcdefghi (5.0%) | 10.58 ± 0.28abcd (2.7%) | 12.39 ± 0.69defghijkl (5.6%) | 21.87 ± 0.49bcdef (2.2%) |
| P3 | 5570.85 ± 940.27ef (16.9%) | 64.07 ± 5.76m (9.0%) | 61.73 ± 5.92ab (9.6%) | 0.55 ± 0.01jk (2.0%) | 0.65 ± 0.19cdefghijk (28.7%) | 5.83 ± 2.26abc (38.9%) | 18.71 ± 1.10abc (5.9%) | 23.68 ± 1.42abcd (6.0%) | 10.69 ± 2.10abcd (19.6%) | 21.64 ± 4.62abc (21.3%) | 40.89 ± 15.37a (37.6%) |
| P4 | 19327.93 ± 6330.63a (32.8%) | 122.34 ± 9.34bcde (7.6%) | 50.14 ± 1.41cde (0.6%) | 0.71 ± 0.01cdefghi (0.2%) | 0.7 ± 0.01cdefghi (0.8%) | 4.66 ± 0.48abc (10.3%) | 6.31 ± 1.92fghijk (30.5%) | 10.51 ± 2.82efghi (26.8%) | 10.96 ± 0.63abcd (5.8%) | 10.82 ± 1.98fghijkl (18.3%) | 21.05 ± 2.27bcdef (10.8%) |
| P5 | 7120.21 ± 478.61cdef (6.7%) | 98.49 ± 0.85defghijk (0.9%) | 38.01 ± 3.80fghi (10.0%) | 0.67 ± 0.04defghijk (6.7%) | 0.65 ± 0.03cdefghijk (4.9%) | 3.76 ± 1.37bc (36.5%) | 9.99 ± 0.71defghij (7.1%) | 13.2 ± 2.75bcdefghi (20.9%) | 8.3 ± 0.75abcd (9.0%) | 14.43 ± 1.22bcdefghijk (8.4%) | 21.81 ± 1.74bcdef (8.0%) |
| P6 | 12052.77 ± 1446.69bc (12.0%) | 125.21 ± 23.75bcd (19.0%) | 63.10 ± 0.92ab (1.5%) | 0.8 ± 0.02bcde (1.9%) | 0.78 ± 0.02bcd (2.8%) | 2.40 ± 0.95c (39.6%) | 1.42 ± 0.92k (29.9%) | 3.91 ± 0.86i (22.0%) | 7.76 ± 1.57bcd (20.2%) | 4.54 ± 0.85l (18.6%) | 12.55 ± 2.19ef (17.5%) |
| P7 | 7601.4 ± 157.42cdef (2.1%) | 108.21 ± 4.07cdefgh (3.8%) | 57.68 ± 5.98abc (10.4%) | 0.77 ± 0.03cdefgh (3.3%) | 0.74 ± 0.02bcdefgh (3.3%) | 7.22 ± 1.31abc (18.2%) | 19.74 ± 0.77ab (3.9%) | 25.83 ± 1.17ab (4.5%) | 9.87 ± 1.26abcd (12.7%) | 20.96 ± 0.86abcd (4.1%) | 29.14 ± 1.32abcde (4.5%) |
| P8 | 11548.01 ± 4628.54bc (40.1%) | 93.71 ± 33.8fghijkl (36.1%) | 59.15 ± 5.97abc (10.1%) | 0.75 ± 0.06cdefghi (7.6%) | 0.72 ± 0.05cdefghi (7.5%) | 3.27 ± 1.72bc (43.4%) | 11.56 ± 5.87cdefghi (33.5%) | 14.48 ± 9.98bcdefghi (34.4%) | 7.09 ± 0.98cd (13.9%) | 15.04 ± 5.13bcdefghij (34.1%) | 21.39 ± 9.15bcdef (19.4%) |
| P9 | 10465.46 ± 1050.4bcde (10.0%) | 89.59 ± 0.71ghijklm (0.8%) | 31.10 ± 1.22hi (3.9%) | 0.62 ± 0.03hijk (4.4%) | 0.58 ± 0.03hijk (5.0%) | 4.27 ± 1.45abc (34.0%) | 4.22 ± 0.38ghijk (9.0%) | 8.53 ± 1.52fghi (17.8%) | 8.32 ± 1.21abcd (14.6%) | 5.45 ± 0.35kl (6.4%) | 13.71 ± 0.75ef (5.5%) |
| P10 | 8855.72 ± 1565cdef (17.7%) | 95.64 ± 1.17efghijk (1.2%) | 34.64 ± 1.08ghi (3.1%) | 0.53 ± 0.04k (6.8%) | 0.50 ± 0.03k (6.0%) | 3.17 ± 0.27bc (8.6%) | 2.04 ± 0.38jk (18.6%) | 5.24 ± 0.20hi (3.8%) | 6.94 ± 0.52cd (7.5%) | 4.57 ± 0.75l (16.4%) | 11.58 ± 0.85f (7.3%) |
| P11 | 12150.46 ± 6798.94bc (39.5%) | 114.53 ± 33.06bcdefg (28.9%) | 43.85 ± 4.23defg (9.7%) | 0.81 ± 0.11bcd (12.9%) | 0.77 ± 0.09bcde (12.2%) | 6.25 ± 2.04abc (32.7%) | 19.34 ± 3.44abc (17.8%) | 24.43 ± 2.74abc (11.2%) | 10.21 ± 1.11abcd (10.9%) | 23.29 ± 3.97ab (17.1%) | 30.53 ± 3.07abcd (10.1%) |
| P12 | 3932.01 ± 1348.86f (34.3%) | 66.23 ± 9.61lm (14.5%) | 45.65 ± 4.38defg (9.6%) | 0.65 ± 0.01ghijk (1.7%) | 0.62 ± 0.01efghijk (2.2%) | 9.55 ± 1.45a (15.1%) | 21.88 ± 1.57a (7.2%) | 29.59 ± 0.50a (1.7%) | 13.58 ± 1.44a (10.6%) | 25.57 ± 1.66a (6.5%) | 35.61 ± 0.86ab (2.4%) |
| P13 | 11773.52 ± 4870.94bc (41.4%) | 130.37 ± 44.4bc (34.1%) | 34.43 ± 7.36ghi (21.4%) | 0.64 ± 0.08hijk (12.4%) | 0.61 ± 0.06fghijk (9.9%) | 6.28 ± 4.28abc (36.3%) | 10.67 ± 12.56ghijk (34.8%) | 16.03 ± 5.30bcdefghi (33.5%) | 9.18 ± 5.14d (13.7%) | 13.65 ± 4.27jkl (16.6%) | 21.73 ± 6.51bcdef (20.8%) |
| P14 | 11668.31 ± 1027.49bc (8.8%) | 101.69 ± 4.99defghij (4.9%) | 44.97 ± 7.93defg (13.1%) | 0.69 ± 0.1cdefghij (14.9%) | 0.66 ± 0.10cdefghij (15.4%) | 7.31 ± 1.55abc (21.2%) | 11.58 ± 2.92cdefgh (25.2%) | 19.67 ± 2.78abcdefg (14.1%) | 12.1 ± 2.78abc (23.0%) | 14.32 ± 3.29bcdefghijk (23.0%) | 26.22 ± 3.76abcdef (14.4%) |
| P15 | 6161.94 ± 899.73def (14.6%) | 80.1 ± 4.11ijklm (5.1%) | 42.41 ± 1.17efg (2.8%) | 0.62 ± 0.02ijk (2.4%) | 0.59 ± 0.01hijk (2.1%) | 3.63 ± 0.38bc (10.4%) | 13.23 ± 0.25bcdef (1.9%) | 16.56 ± 0.17bcdefghi (1.0%) | 7.86 ± 0.89bcd (11.3%) | 15.85 ± 0.31bcdefgh (1.9%) | 22.57 ± 0.44bcdef (2.0%) |
| P16 | 10159.2 ± 1939.55bcde (19.1%) | 93.36 ± 0.96fghijkl (1.0%) | 44.34 ± 3.88defg (8.8%) | 0.61 ± 0.01ijk (2.2%) | 0.6 ± 0.02ghijk (3.6%) | 4.23 ± 0.84abc (20.0%) | 9.17 ± 0.77defghijk (8.4%) | 13.04 ± 1.43bcdefghi (11.0%) | 10.57 ± 2.36abcd (22.4%) | 13.11 ± 0.68cdefghijkl (5.2%) | 22.59 ± 2.43bcdef (10.8%) |
| P17 | 8607.04 ± 1378.67cdef (16.0%) | 90.86 ± 10.03fghijklm (11.0%) | 38.98 ± 9.75fgh (25.0%) | 0.64 ± 0.05hijk (7.4%) | 0.6 ± 0.03ghijk (5.1%) | 6.02 ± 1.41abc (23.4%) | 4.89 ± 0.49ghijk (10.1%) | 10.79 ± 1.64defghi (15.2%) | 8.74 ± 0.71abcd (8.2%) | 7.03 ± 0.71hijkl (10.1%) | 15.12 ± 0.87def (5.7%) |
| P18 | 5995.13 ± 2122.72def (35.4%) | 88.48 ± 34.93ghijklm (39.5%) | 34.72 ± 16.84ghi (16.8%) | 0.61 ± 0.2ijk (32.6%) | 0.58 ± 0.18ijk (31.1%) | 4.43 ± 0.69abc (15.6%) | 8.09 ± 1.85efghijk (22.9%) | 12.46 ± 1.9cdefghi (15.2%) | 8.37 ± 0.84abcd (10.0%) | 10.89 ± 2.38fghijkl (21.8%) | 18.87 ± 2.60bcdef (13.8%) |
| P19 | 11917.79 ± 536.03bc (4.5%) | 125.35 ± 0.89bcd (0.7%) | 62.29 ± 4.95ab (7.9%) | 0.80 ± 0.04bcdef (4.5%) | 0.76 ± 0.03bcdef (3.3%) | 3.78 ± 0.41bc (10.8%) | 3.77 ± 0.78hijk (20.6%) | 7.44 ± 0.69ghi (9.3%) | 8.44 ± 1.18abcd (14.0%) | 6.86 ± 0.84ijkl (12.2%) | 14.84 ± 1.78def (12.0%) |
| P20 | 5142.44 ± 1975.83ef (38.4%) | 75.96 ± 16.6jklm (21.9%) | 36.73 ± 4.69fghi (12.8%) | 0.68 ± 0.03cdefghijk (4.3%) | 0.64 ± 0.02defghijk (3.5%) | 4.61 ± 0.19abc (4.1%) | 8.74 ± 1.82efghijk (20.8%) | 13.46 ± 1.8bcdefghi (13.3%) | 7.99 ± 0.40bcd (5.0%) | 11.7 ± 1.32efghijkl (11.3%) | 18.94 ± 1.23bcdef (6.5%) |
| P21 | 6946.31 ± 2312.40cdef (33.3%) | 74.94 ± 11.31jklm (15.1%) | 27.74 ± 0.99i (3.6%) | 0.62 ± 0.11hijk (18.4%) | 0.6 ± 0.09ghijk (15.6%) | 6.89 ± 1.14abc (16.6%) | 17.04 ± 2.54abcd (14.9%) | 22.9 ± 2.28abcde (10.0%) | 10.53 ± 0.91abcd (8.6%) | 20.48 ± 2.47abcde (12.0%) | 28.89 ± 1.86abcde (6.4%) |
| P22 | 5840.84 ± 2170.03def (37.2%) | 118.13 ± 10.73bcdef (9.1%) | 35.84 ± 2.05fghi (5.7%) | 0.82 ± 0.03bc (3.5%) | 0.77 ± 0.03bcde (4.0%) | 8.21 ± 1.04ab (12.6%) | 19.11 ± 2.74abc (14.3%) | 25.89 ± 3.23ab (12.5%) | 11.78 ± 0.94abc (7.9%) | 21.75 ± 3.06abc (14.1%) | 31.19 ± 3.68abcd (11.8%) |
| P23 | 5327.27 ± 416.95ef (7.8%) | 84.66 ± 7.74hijklm (9.1%) | 43.61 ± 2.89defg (6.6%) | 0.66 ± 0.05efghijk (8.3%) | 0.63 ± 0.04defghijk (6.3%) | 7.05 ± 3.03abc (42.9%) | 14.65 ± 2.89abcde (19.8%) | 21.06 ± 4.16abcdef (19.7%) | 9.86 ± 3.82abcd (28.6%) | 15.73 ± 3.97bcdefghi (25.2%) | 24.76 ± 5.79abcdef (23.4%) |
| P24 | 14975.54 ± 1736.28ab (11.6%) | 167.26 ± 11.88a (7.1%) | 57.99 ± 5.11abc (10.7%) | 0.93 ± 0.04ab (4.5%) | 0.88 ± 0.04ab (4.3%) | 2.60 ± 1.65c (40.4%) | 9.82 ± 0.93defghij (9.5%) | 12.47 ± 2.05cdefghi (16.4%) | 6.89 ± 1.29cd (18.7%) | 12.61 ± 0.76defghijkl (6.0%) | 18.78 ± 0.73cdef (3.9%) |
| P25 | 7072.25 ± 209.54cdef (3.0%) | 80.74 ± 1.64hijklm (2.0%) | 41.28 ± 4.28efgh (10.4%) | 0.54 ± 0.01jk (1.3%) | 0.52 ± 0.01jk (2.7%) | 2.74 ± 0.38c (14.0%) | 8.98 ± 2.49defghijk (27.7%) | 11.5 ± 2.04cdefghi (17.8%) | 7.55 ± 0.38cd (5.0%) | 13.8 ± 1.37cdefghijk (9.9%) | 18.87 ± 3.19bcdef (16.9%) |
| P26 | 7082.18 ± 1349.10cdef (19.0%) | 113.49 ± 23.17bcdefg (20.4%) | 53.82 ± 4.99bcd (9.3%) | 0.79 ± 0.07bcdefg (9.0%) | 0.76 ± 0.07bcdefg (9.3%) | 4.93 ± 0.49abc (9.8%) | 13.45 ± 0.40bcdef (2.9%) | 17.86 ± 0.42abcdefgh (2.3%) | 8.46 ± 1.37abcd (16.1%) | 16.77 ± 1.25abcdefg (7.5%) | 24.34 ± 1.83abcdef (7.5%) |
| P27 | 11693.26 ± 720.77bc (6.2%) | 138.27 ± 16.05b (11.6%) | 64.95 ± 3.49a (5.4%) | 0.97 ± 0.26a (12.0%) | 0.92 ± 0.25a (26.7%) | 8.51 ± 1.08bc (40.4%) | 15.83 ± 3.91fghijk (28.4%) | 23.1 ± 2.68fghi (41.9%) | 12.98 ± 1.36ab (10.5%) | 18.53 ± 3.68abcdef (19.9%) | 34.19 ± 6.74abc (19.7%) |
| P28 | 7238.80 ± 2717.40cdef (37.5%) | 71.34 ± 2.60klm (3.6%) | 38.59 ± 6.31fghi (16.4%) | 0.64 ± 0.05hijk (8.1%) | 0.60 ± 0.05ghijk (8.6%) | 6.14 ± 3.55abc (41.5%) | 4.34 ± 0.33ghijk (7.7%) | 10.15 ± 3.76efghi (37.0%) | 11.23 ± 3.94abc (26.2%) | 6.66 ± 0.57jkl (8.6%) | 17.44 ± 4.06cdef (23.3%) |
| P29 | 12110.03 ± 4056.50bc (33.5%) | 129.80 ± 14.55bc (11.2%) | 46.65 ± 7.49def (16.0%) | 0.75 ± 0.05cdefghi (6.8%) | 0.71 ± 0.05cdefghi (7.6%) | 3.32 ± 1.34ab (12.7%) | 5.65 ± 2.61abcde (24.7%) | 8.9 ± 3.73abcde (11.6%) | 6.85 ± 1.09cd (15.9%) | 8.05 ± 2.21ghijkl (27.4%) | 14.58 ± 2.55def (17.5%) |
| P30 | 10160.61 ± 5092.10bcde (40.3%) | 88.88 ± 2.97ghijklm (3.3%) | 43.60 ± 3.58defg (8.2%) | 0.65 ± 0.04fghijk (6.8%) | 0.63 ± 0.05defghijk (8.6%) | 6.58 ± 1.81abc (27.6%) | 9.33 ± 4.48defghijk (26.5%) | 15.65 ± 5.55bcdefghi (35.5%) | 9.09 ± 2.31abcd (25.5%) | 12.33 ± 5.05defghijkl (16.7%) | 20.85 ± 6.80bcdef (23.0%) |
| P31 | 11136.26 ± 1249.15bcd (11.2%) | 140.05 ± 4.48b (3.2%) | 42.39 ± 5.54efg (13.1%) | 0.82 ± 0.02bc (2.8%) | 0.80 ± 0.03abc (3.9%) | 3.64 ± 1.09bc (30.0%) | 3.41 ± 1.56ijk (31.2%) | 7.23 ± 2.46ghi (34.0%) | 8.98 ± 2.09abcd (23.3%) | 6.65 ± 1.80jkl (27.1%) | 15.37 ± 2.50def (16.3%) |

**Note.** Values are means ± standard deviations, with coefficients of variation (CV, %) in parentheses. Different lowercase superscript letters within a column indicate significant differences among provenances at *P* < 0.05. MOE, modulus of elasticity; MOR, modulus of rupture; CS, compressive strength parallel to grain.
