# Supplementary material for: Geographic variation in Dendrocalamus latiflorus culm physical and mechanical traits is associated with source-climate gradients
Source: Front Plant Sci. 2026 Oct 14;17:1946733. doi: 10.3389/fpls.2026.1946733 (PMC13713850; doi:10.3389/fpls.2026.1946733)
Supplement: Supplementary file 2 [file SupplementaryFile2.docx]

Supplementary Material

**Supplementary Table S4.** Unified comparison of 11 culm physical and mechanical traits in selected high-performing provenances.

| **Provenance** | **MOE (MPa)** | **MOR (MPa)** | **CS (MPa)** | **ADD (g cm−3)** | **ODD (g cm−3)** | **ADTS (%)** | **ADRS (%)** | **ADVS (%)** | **ODTS (%)** | **ODRS (%)** | **ODVS (%)** |
| --- | --- | --- | --- | --- | --- | --- | --- | --- | --- | --- | --- |
| P4 | 19327.93 ± 6330.63 | 122.34 ± 9.34 | 50.14 ± 1.41 | 0.71 ± 0.01 | 0.70 ± 0.01 | 4.66 ± 0.48 | 6.31 ± 1.92 | 10.51 ± 2.82 | 10.96 ± 0.63 | 10.82 ± 1.98 | 21.05 ± 2.27 |
| P6 | 12052.77 ± 1446.69 | 125.21 ± 23.75 | 63.10 ± 0.92 | 0.80 ± 0.02 | 0.78 ± 0.02 | 2.40 ± 0.95 | 1.42 ± 0.92 | 3.91 ± 0.86 | 7.76 ± 1.57 | 4.54 ± 0.85 | 12.55 ± 2.19 |
| P19 | 11917.79 ± 536.03 | 125.35 ± 0.89 | 62.29 ± 4.95 | 0.80 ± 0.04 | 0.76 ± 0.03 | 3.78 ± 0.41 | 3.77 ± 0.78 | 7.44 ± 0.69 | 8.44 ± 1.18 | 6.86 ± 0.84 | 14.84 ± 1.78 |

*Note.* Values are means ± standard deviations. P6, P4, and P19 were the three highest-ranked provenances in the revised seven-criterion entropy-weighted TOPSIS analysis. ADD/ODD, air-dry/oven-dry density; ADTS/ADRS/ADVS, air-dry tangential/radial/volumetric shrinkage; ODTS/ODRS/ODVS, oven-dry tangential/radial/volumetric shrinkage.

**Supplementary Table S5.** Objective validation of candidate Ward cluster solutions.

| **Number of clusters (k)** | **Silhouette coefficient** | **Calinski-Harabasz index** | **Davies-Bouldin index** | **Decision** |
| --- | --- | --- | --- | --- |
| 2 | 0.317 | 14.87 | 1.180 | Selected |
| 3 | 0.282 | 13.89 | 1.123 |  |
| 4 | 0.217 | 12.72 | 1.303 |  |
| 5 | 0.237 | 12.91 | 1.217 |  |
| 6 | 0.255 | 12.32 | 1.095 |  |

*Note.* The two-cluster solution was selected because it maximized both the silhouette coefficient and Calinski-Harabasz index among k = 2–6. Lower Davies-Bouldin values are preferable, but this metric alone did not override the joint evidence from the other validation indices and the dendrogram structure.

**Supplementary Table S6.** Sensitivity of TOPSIS rankings to entropy versus equal weighting.

| **Provenance** | **Entropy-weight Ci** | **Entropy rank** | **Equal-weight Ci** | **Equal-weight rank** | **Rank change** |
| --- | --- | --- | --- | --- | --- |
| P6 | 0.781 | 1 | 0.727 | 2 | 1 |
| P4 | 0.771 | 2 | 0.712 | 3 | 1 |
| P19 | 0.739 | 3 | 0.698 | 4 | 1 |
| P31 | 0.720 | 4 | 0.667 | 5 | 1 |
| P29 | 0.717 | 5 | 0.661 | 6 | 1 |
| P24 | 0.695 | 6 | 0.749 | 1 | -5 |
| P10 | 0.685 | 7 | 0.547 | 8 | 1 |
| P9 | 0.672 | 8 | 0.544 | 9 | 1 |
| P17 | 0.601 | 9 | 0.511 | 10 | 1 |
| P28 | 0.578 | 10 | 0.470 | 16 | 6 |
| P8 | 0.567 | 11 | 0.551 | 7 | -4 |
| P16 | 0.566 | 12 | 0.483 | 15 | 3 |
| P25 | 0.551 | 13 | 0.446 | 18 | 5 |
| P13 | 0.533 | 14 | 0.509 | 11 | -3 |
| P18 | 0.517 | 15 | 0.430 | 22 | 7 |
| P5 | 0.514 | 16 | 0.443 | 20 | 4 |
| P30 | 0.513 | 17 | 0.468 | 17 | 0 |
| P2 | 0.513 | 18 | 0.486 | 14 | -4 |
| P1 | 0.503 | 19 | 0.500 | 12 | -7 |
| P20 | 0.486 | 20 | 0.414 | 24 | 4 |
| P14 | 0.430 | 21 | 0.442 | 21 | 0 |
| P15 | 0.425 | 22 | 0.375 | 25 | 3 |
| P26 | 0.412 | 23 | 0.444 | 19 | -4 |
| P27 | 0.351 | 24 | 0.491 | 13 | -11 |
| P11 | 0.330 | 25 | 0.416 | 23 | -2 |
| P23 | 0.308 | 26 | 0.316 | 27 | 1 |
| P21 | 0.259 | 27 | 0.247 | 30 | 3 |
| P7 | 0.230 | 28 | 0.354 | 26 | -2 |
| P3 | 0.203 | 29 | 0.250 | 29 | 0 |
| P22 | 0.183 | 30 | 0.295 | 28 | -2 |
| P12 | 0.076 | 31 | 0.157 | 31 | 0 |

*Note.* Spearman rank correlation between the two schemes was ρ = 0.911 (P < 0.001). Seven of the eight entropy-weight upper-quartile provenances also occurred in the equal-weight upper quartile. Positive rank change indicates a lower position under equal weighting.
